# Axo-axonic cells in neuropsychiatric disorders: a systematic review

**DOI:** 10.3389/fncel.2023.1212202

**Published:** 2023-06-26

**Authors:** Juliette Vivien, Anass El Azraoui, Cloé Lheraux, Frederic Lanore, Bruno Aouizerate, Cyril Herry, Yann Humeau, Thomas C. M. Bienvenu

**Affiliations:** ^1^Université de Bordeaux, Inserm Neurocentre Magendie U1215, Bordeaux, France; ^2^Univ Bordeaux, CNRS, Interdisciplinary Institute for Neuroscience, IINS, UMR 5297, Bordeaux, France; ^3^Centre Hospitalier Charles Perrens, Inserm Neurocentre Magendie U1215, Bordeaux, France; ^4^INRAE, Bordeaux INP, NutriNeuro, UMR 1286, Bordeaux, France

**Keywords:** interneuron, chandelier cell, epilepsy, schizophrenia, autism

## Abstract

Imbalance between excitation and inhibition in the cerebral cortex is one of the main theories in neuropsychiatric disorder pathophysiology. Cortical inhibition is finely regulated by a variety of highly specialized GABAergic interneuron types, which are thought to organize neural network activities. Among interneurons, axo-axonic cells are unique in making synapses with the axon initial segment of pyramidal neurons. Alterations of axo-axonic cells have been proposed to be implicated in disorders including epilepsy, schizophrenia and autism spectrum disorder. However, evidence for the alteration of axo-axonic cells in disease has only been examined in narrative reviews. By performing a systematic review of studies investigating axo-axonic cells and axo-axonic communication in epilepsy, schizophrenia and autism spectrum disorder, we outline convergent findings and discrepancies in the literature. Overall, the implication of axo-axonic cells in neuropsychiatric disorders might have been overstated. Additional work is needed to assess initial, mostly indirect findings, and to unravel how defects in axo-axonic cells translates to cortical dysregulation and, in turn, to pathological states.

## Introduction

Imbalance between synaptic excitation and inhibition in cortical regions (including neocortex and hippocampus) is a key feature of many brain disorders (Marin, 2012; Sohal and Rubenstein, 2019). Activities of cortical neuron assemblies are coordinated by brain rhythms of various frequencies which are known to be critical for cognitive, emotional and motor function (Buzsaki and Draguhn, 2004). Throughout the brain, inhibition provided by GABAergic interneurons orchestrate cortical oscillations (Roux and Buzsaki, 2015). Lack of pyramidal neuron inhibition results in global hyperexcitability and highly synchronous discharges, which are hallmarks of epilepsy (EPI). Beyond hyperexcitability, dysregulation of oscillatory activities may result in more subtle alterations in cortical functions. Basal and sensory-evoked oscillatory rhythms have been found to be altered in schizophrenia (SCZ) and autism spectrum disorders (ASD; Simon and Wallace, 2016; Hunt et al., 2017). As inhibition is critical for cortical oscillations (Roux and Buzsaki, 2015), these findings collectively point toward a central implication of interneuron functional alterations in neuropsychiatric disorders (Marin, 2012; Sohal and Rubenstein, 2019).

In cortex, inhibition is provided by distinct types of GABAergic cells, with extensive diversity in structure reflected in specialized functions. Indeed, by receiving different synaptic inputs and by targeting distinct functional domains along somato-dendritic arborizations of pyramidal cells, distinct GABAergic interneuron types are thought to play highly specific roles in organizing cortical network activities (e.g., Kepecs and Fishell, 2014). Perisomatic inhibition is mainly provided by interneurons which typically emit short-duration action potentials at high frequencies and express the calcium binding protein parvalbumin (PV). PV+ interneurons are powerful inhibitors of principal neurons and regulate fast (>30 Hz) brain rhythms, such as gamma oscillations (30–80 Hz; Cardin et al., 2009; Sohal et al., 2009). This, added to their expression of disease-related genes, has led to the hypothesis that PV+ interneuron dysfunction may be implicated in neuropsychiatric disorders (reviewed in Marin, 2012).

The PV+ interneuron class has been further divided into two main types: basket cells, which axon make synapses with cell bodies and proximal dendrites of pyramidal neurons, and axo-axonic cells (AACs), which axonal boutons exclusively innervate the axon initial segment (AIS) of pyramidal neurons (Somogyi, 1977). Axo-axonic terminals are organized in vertical clusters (“cartridges”), hence the alternative name of “Chandelier cells” for AACs. This typical morphology is the most distinctive characteristic of AACs at the cellular level. In addition, PV is a classical (albeit non-selective) marker of AACs (but PV- AACs are found in cortex, e.g., Taniguchi et al., 2013). In addition, Human AACs may express calbindin (CB), which has been found to be associated (del Rio and DeFelipe, 1997), or mutually exclusive with PV (Wittner et al., 2002). At the electron microscopic level, AACs make symmetrical synapses with the axon initial segment (identified by cisternal organelles, dense undercoating, bundled microtubules). Accordingly, axo-axonic terminals contain the molecular machinery for GABA release, including glutamic acid decarboxylase (GAD), membrane GABA transporters (GAT-1) and vesicular GABA transporters (VGAT; Jung et al., 2022). At the postsynaptic level, AISs are organized by scaffold proteins with Ankyrin-G playing a decisive role (Gallo et al., 2020). AISs synapses are enriched in α2 subunit-containing GABA_A_-Rs (Nusser et al., 1996), although a quantitative freeze-fracture replica study found equal levels of α1 and α2 subunits (Kerti-Szigeti and Nusser, 2016). These morphological and molecular features are classically used to study AACs, including in the studies reviewed in this article.

Because action potentials are generated at the AIS, AACs have long been speculated to exert powerful control on pyramidal neurons activities, and to play a key role in regulating network excitability and neuronal assemblies. Being sole inhibitors at the AIS and showing disease-related changes, AACs might represent a cortical Achille's heel. Indeed, the potential role of AACs in network physiology has been mirrored by studies of cortical AACs in disease, pointing toward abnormalities at AACs' cell bodies and synapses in postmortem samples of patients suffering from EPI (DeFelipe et al., 1993), SCZ (Woo et al., 1998) or, more recently, ASD (Ariza et al., 2018). AACs and their implication in disease have attracted a lot of interest, as previously summarized (Wang et al., 2016; Gallo et al., 2020; Juarez and Martinez Cerdeno, 2022). However, no previous work has reviewed *systematically* the studies linking AACs to brain disorders. The aim of this article is to provide a systematic review of articles documenting AACs' implication in neuropsychiatric disorders (EPI, SCZ, and ASD), for scientists and clinicians studying those disorders, and to help design future studies on robust experimental grounds.

## Methods

The systematic review followed PRISMA guidelines. Two independent electronic databases (Pubmed and Web of Science) were searched until July 2021 for articles dealing with AACs in EPI, SCZ and ASD with the following search terms: ≪(chandelier cell OR axo axonic) AND epilep^*^≫, ≪(chandelier cell OR axo axonic) AND schizophrenia≫, ≪(chandelier cell OR axo axonic) AND autism≫, respectively. Duplicates were removed, and titles and abstracts were screened for inclusion. Full texts were obtained and examined if articles potentially corresponded to eligibility criteria. Two investigators (AEA and JV) screened the literature. In case of a disagreement, individual articles were studied by all the authors, until consensus was reached. Full articles matching inclusion criteria were studied and summarized by the authors. Additional articles found by cross-referencing were also examined and included when indicated.

Eligibility was defined by the following criteria, which were defined prior to the selection of articles:

### Inclusion criteria

Articles investigating AACs' modifications in EPI, SCZ, ASD in brain tissue from patients (including case reports), or in animal models of those disorders (*in vivo* and/or *in vitro*) were included for analysis.

### Exclusion criteria

Articles written in languages other than English, review articles, and studies giving no specific results on AACs were excluded from the final analysis. Articles lacking an unambiguous (e.g., morphological) identification of AACs or their synaptic compartments at AISs were thus excluded. Likewise, studies lacking a characterization of potential AACs' alterations were excluded. All exclusions were documented and reasons for exclusions are reported.

## Results

**Figure 1:**
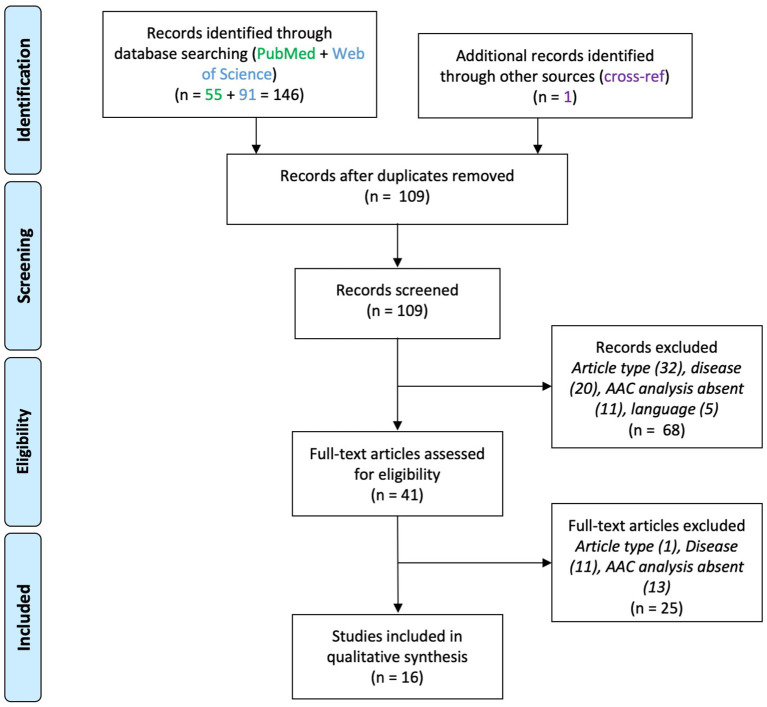
PRISMA flowchart for studies on axo-axonic cells in epilepsy.

**Figure 2:**
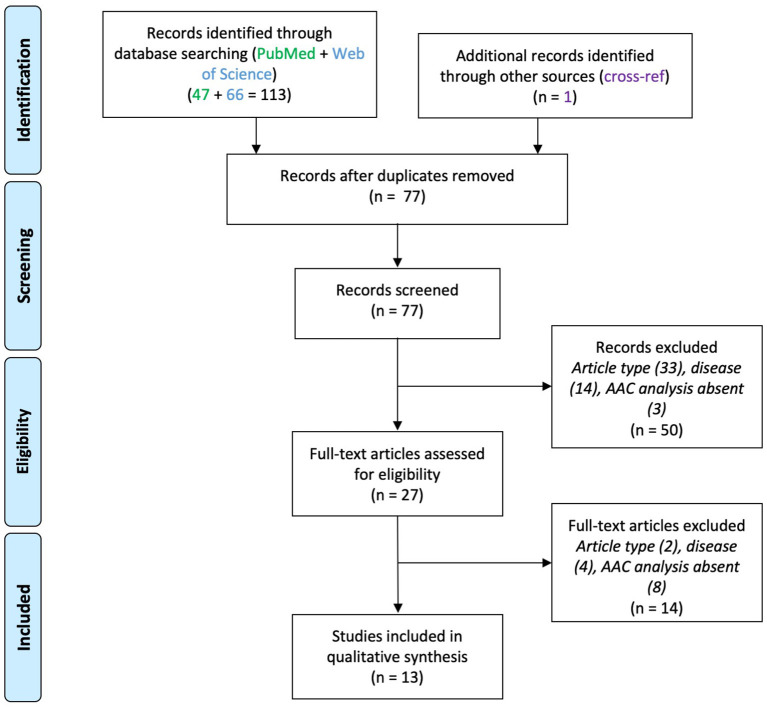
PRISMA flowchart for studies on axo-axonic cells in schizophrenia.

**Figure 3:**
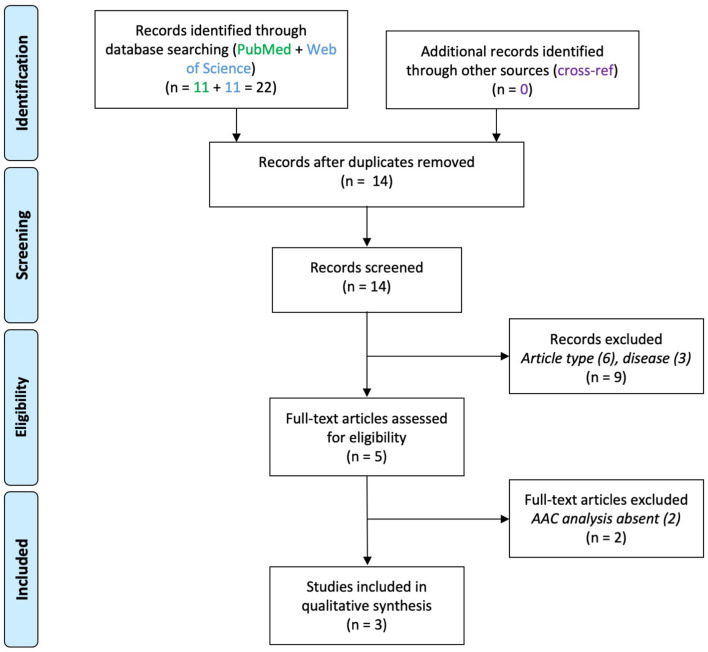
PRISMA flowchart for studies on axo-axonic cells in autism spectrum disorder.

A total of 200 articles were screened after duplicates removal (EPI 109, SCZ 77, ASD 14). Thirty-two articles were included in the final analysis (EPI 16, SCZ 13, ASD 3). The screening steps are illustrated in the corresponding flowcharts (Figures 1–**3**; see also Supplementary material). The results concerning AACs are summarized in Tables 1–**3**.

**Table 1:** Summary of studies on axo-axonic cells in epilepsy.

**References**	**Model(s)**	**Population characteristics (ratio M:F; age range)**	**Area(s) studied**	**Methods**	**Variable(s) studied**	**Main findings**	**Comments**
Ribak (1985)	Monkey Alumina gel-injected monkeys(intracortical or sub-arachoid)	Three adult monkeys (*Macaca mulatta* & *M. fascicularis*)	Sensorimotor cortex, layers 2, 3 and 5	Electron microscopy	Number of AACs boutons contacting the AIS	Decreased number of AACs boutons at the level of the AIS (from 10 to 15 terminals per AIS in control hemisphere to 0–3 in epileptic loci).	Semi-quantitative study (only ranges provided). Age and gender of monkeys not known.
Best et al. (1994)	KA-lesioned Wistar rats (ICV injections)	Juvenile rats (Wistar, 180 g), all male. 15 M injected and 5 M; age-matched controls.	Hippocampus (CA1)	PV IHC, light and electron microscopy	Distribution of PV+ axonal boutons 3 days, 1, 2, 4 weeks and 3 months.	No degenerating PV+ AAC boutons at the AIS of pyramidal cells in KA-treated hemispheres, at any time point vs. almost total loss of PV+ perisomatic innervation.	Qualitative study, small sample size.
Sloviter et al. (2003)	Perforant path electrically-stimulated and KA-lesioned rats (IV)	Young adult rats (Sprague-Dawley, 250–400 g), all male: (PP-stimulation *n* = 4; KA: *n* = 2)	Hippocampus (Dentate gyrus)	Electron microscopy	Degeneration of AAC boutons at days 4 and 7 post PP stimulation or 10 post-KA injection	No degeneration of axo-axonic boutons in kainic acid treated rats, nor in PP-stimulated rats.	Qualitative study, with exclusive use of degenerating axonal profiles as a marker of AAC loss/damage.
Dinocourt et al. (2003)	Pilocarpine-treated rats (IP injection)	Young adult rats (Wistar, 200–290 g), all males (pilocarpine: *n* = 10 M; seven for light microscopy, three for electron microscopy vs. three matched controls)	Hippocampus (CA1 stratum oriens)	Light (with PV and SOM IHC) and electron microscopy.	Density of AACs boutons contacting the AIS of pyramidal cells at 3 months post-treatment	Decrease in synaptic density and synaptic coverage at the AIS in CA1 pyramidal cells, of pilocarpine treated rats (both of 63%). And 34% decrease in number of PV-expressing, somatostatin-negative (putative AAC) in str. oriens compared to controls.	Limited cohort for the electron microscopic study of synapses at the AIS (three controls + three pilocarpine-treated)
Karlócai et al. (2014)	High potassium-treated brain slices of mice (epileptic discharges).	Juvenile mice (P19–P40), wild type (CD1 and BL6 strains) and transgenic (PV-eGFP) of both genders	Hippocampus (CA3)	Local field potential combined with intracellular recording (loose-patch, whole-cell) followed by anatomical identification of the recorded neurons	AAC firing around and during epileptiform events, compared to sharp wave ripples.	Increase in firing rates during epileptiform events, compared to spontaneously occurring sharp wave-ripples.	*In vitro* model without *in vivo* measurements.
Ferrer et al. (1992)	Surgical resection tissue in a patient suffering from drug-resistant epilepsy due to cortical dysplasia	1F; 14-year-old (cortical dysplasia)	Peri-rolandic region (primary motor and primary somatosensory cortices)	PV IHC	Morphology of PV+ AACs in dysplastic tissue	PV immunoreactivity is decreased in areas of tissue necrosis whereas neighboring dysplastic areas display abnormal populations of very-large PV+ AACs.	Qualitative, single-case study, of a particular condition (dysplasia). Only one abnormal AAC described in the body of the article.
DeFelipe et al. (1993)	Peri-lesional surgical resection tissue from drug-resistant patients with TLE	2:3; 12–34 yrs (intractable TLE)	BA20, 21 and 38	EcoG. PV IHC. Light and electron microscopy	Density of PV+ AAC cartridges in epilepsy	Heterogeneous patterns, with 2/4 patterns showing a global loss of PV+ axon and 1/4 showing a specific loss of PV+ AAC cartridges (uncorrelated with the EcoG spiking profile).	Qualitative study (no quantification, no control). Quantification of AAC terminals only in pattern “A” (“normal”). Only PV+ AACs were studied.
Ferrer et al. (1994)	Surgical resection tissue from patients affected by different forms of epilepsy	17:13; 9–50 yrs one diffuse hypoxic encephalopathy, nine neoplasms, two focal cortical dysplasia, one with subcortical ectopic neuronal nests, one with focal disorder of migration, two cavernous angiomas, 12 temporal lobe epilepsy with mesial sclerosis, two cryptogenic epilepsy	Various epileptic foci including frontal and temporal regions.	PV IHC; light microscopy	Density and morphology of PV+ axons	Increase in number and size of PV+ AACs cartridges in some discrete areas (hypertrophic cartridges) in the only case for which AACs analysis is reported, of diffuse hypoxic encephalopathy.	Qualitative study without controls, epilepsy is associated with another disorder. Result about AAC is from only one patient. Only PV+ AACs are studied.
Marco et al. (1996)	Peri-lesional surgical resection tissue from patients with TLE (±tumors)	6:3; 13–44 (epileptic without brain tumor); 2:2; 18–30 (epileptic with brain tumors); 2 M; 70–91 (controls)	BA20, 21, and 38	EcoG. PV IHC	Density of PV immunostaining and PV+ AAC cartridges	Heterogeneous patterns, with 2/4 patterns showing a global loss of PV+ axon and 1/4 showing a specific loss of PV+ AAC cartridges (both uncorrelated with the EcoG profile). NB: AAC alterations present in 5/9 samples of TLE without tumor, and 4/4 of TLE with tumor)	Qualitative study. Only 2 controls (older). Only PV+ AACs are studied.
Marco et al. (1997)	Peri-lesional surgical resection tissue from one patient with secondary TLE (astrocytoma)	1F; 30-year-old (epilepsy and brain tumor); tissue sections from various controls	BA38	PV IHC. Light and electron microscopy.	PV+ cells and axon terminals. Synapses at the AIS.	Decreased in AACs' cartridges density. Decreased number of synapses at the AIS accompanied by an increased gliosis.	Qualitative study. Case report from an epileptic patient with a brain tumor, who underwent radiotherapy and previous surgical treatments.
Wittner et al. (2001)	Surgical resection tissue from patients with TLE (with hippocampal sclerosis)	14:9; 17–56 (intractable TLE with 3 degrees of sclerosis); 3:3; 37–56 (controls)	Hippocampus (dentate gyrus)	PV IHC. Light and electron microscopy	Number of PV+ boutons contacting the AIS (LM) and synaptic density at the AIS (EM).	Increased density of synapses at the AIS of granule cells, following degrees of hippocampal sclerosis. NB: loss or decrease of PV immunoreactivity at light and electron microscopic levels.	Semi-quantitative study (no statistical analysis).
Wittner et al. (2002)	Surgical resection tissue from patients with TLE (hippocampal sclerosis)	14:9; 17–56 (intractable TLE with mild−6 samples- or strong sclerosis−17 samples-); 3:3; 37–56 (controls)	Hippocampus (CA1)	CB IHC. Light and electron microscopy	Subcellular target distribution of CB-positive axon terminals	The proportion of AISs among the target elements of CB+ terminals considerably decreased or even disappeared in the strongly sclerotic cases.	Same cohort as Wittner et al. (2001).
Arellano et al. (2004)	Surgical resection tissue from patients with TLE	8:6; 17–50 (TLE with hippocampal sclerosis); 3 M; 23–63 (controls)	Hippocampus (dentate gyrus, CA, subiculum)	GAT1, CB, PV, NeuN and PSA-NCAM IHC; light microscopy	Morphology and density of AAC cartridges.	Increased complexity of cartridges on surviving granule cells (dentate gyrus) and pyramidal cells (CA). No difference in subiculum. NB: general reduction in CB & PV immunoreactivity.	Qualitative study. Regional heterogeneity within individual samples and across patients
Alonso-Nanclares et al. (2005)	Surgical resection tissue from patients with focal cortical dysplasia	1:2; 3.5–24 (focal cortical dysplasia); 3 M; 24–37 epileptic patients with hippocampal sclerosis (controls)	Different location depending on the site of lesion (parietal lobe, fronto-insular cortex or inferior frontal gyrus)	PV IHC. Light and electron microscopy	PV+ axon terminals.	Decreased density of PV+ cartridges and/or of immunoreactivity in cartridges on giant neurons. Decrease in axo-axonic innervation is accompanied by an increase in axo-somatic innervation.	Qualitative study. Epilepsy associated with FCD. Only three patients with the youngest being 3.5 years old. Regional heterogeneity within individual samples and across patients. Controls also suffer from seizures, with hippocampal sclerosis. Only PV+ AACs are studied. No electron microscopic report of axon initial segment innervation.
Wittner et al. (2005)	Biopsy brain tissues from patients with TLE	17:15; 14–56 (TLE; four grades: mild cell loss, patchy cell loss, sclerosis, gliosis); 4:3; 37–56 (controls)	Hippocampus (CA1)	PV IHC. Light and electron microscopy.	PV+ cells and axons density (LM); synaptic coverage of AIS (EM)	No significant difference in AIS synaptic innervation (EM, presynapses containing or not PV). Decreased synaptic coverage of degenerating AISs. Severe PV+ cell loss in patchy hippocampal cell loss and sclerosis cases, with no axonal staining for PV in the sclerotic CA1. No difference in cases of mild cell loss.	Observation of degenerating AISs limited to two cases (qualitative observation). Same control group as Wittner et al. (2001, 2002), except one case.
Alhourani et al. (2020)	Biopsy brain tissues from Patients with TLE	3:2; 37–49 (intractable TLE); 3:2, 36–50 (matched controls)	Hippocampus (dentate gyrus)	IHC: VGAT, PV, GAD65 and GAD67. Quantitative fluorescent confocal microscopy	Density of AAC boutons (vGAT+/GAD67+/ GAD65-) and GAD67/vGAT/PV protein levels in these boutons.	No change in VGAT+/GAD67+ bouton density Decreased PV+/VGAT+ bouton density in the sclerotic group (92%); no difference between non-sclerotic and control samples Increased levels of GAD67 and vGAT is in the sclerotic group compared to both groups (GAD67: vs. control: +482%; vs. non-sclerotic: +212%; vGAT: +400–500%).	Quantitative study Matched controls No report of density of VGAT+/GAD67+ boutons per tissue volume (only normalized by total neuron number)

BA, Brodmann area; CA, *Cornu Ammonis* (hippocampal region); CB, calbindin; ECoG, electrocorticography; EGFP, enhanced green fluorescent protein; EM, electron microscopy; GAD(65, 67), glutamic acid decarboxylase (65/67 kDa isoforms); GAT1, GABA transporter type1; ICV, intracerebroventricular (injection); IHC, immunohistochemistry (also used here for immunofluorescence for simplicity); IP, intraperitoneal (injection); IV, intravenous (injection); KA, kainic acid; LM, light microscopy; NeuN, neuronal nuclei (marker); PSA-NCAM, polysialylated form of neural cell adhesion molecule; PV, parvalbumin; SOM, somatostatin; TLE, temporal lobe epilepsy; VGAT, vesicular GABA transporter.

### Epilepsy

Sixteen papers were included (Figure 1). These studies used either human brain samples from patients affected by medically intractable epilepsy (*n* = 11) or from animal models of epilepsy (*n* = 5). Neocortical and hippocampal alterations in AACs were reported, which are divided below for clarity. Results are summarized in Table 1.

#### Neocortex

In the neocortex, a loss of PV+ AACs' cartridges has been reported in the anterior temporal lobe of epileptic patients, with some samples displaying pathological cytoarchitectures (DeFelipe et al., 1993; Marco et al., 1996). The decrease in PV+ AACs' cartridges has also been observed in dysplastic samples from patients suffering from EPI secondary to focal cortical dysplasia (Alonso-Nanclares et al., 2005). In a case report of a young patient with cortical dysplasia, a decrease in PV immunoreactivity was described (Ferrer et al., 1992), suggesting that the decreased density of cartridges observed in the previous studies may correspond to a decrease in PV levels. This finding was associated with abnormal morphologies of the remaining PV+ cells, including the observation of an enlarged AAC (Ferrer et al., 1992). Ferrer and colleagues also reported hypertrophic PV+ cartridges in a patient suffering from diffuse hypoxic encephalopathy associated with focal seizures (Ferrer et al., 1994). A case report of a patient suffering from seizures secondary to a temporal lobe astrocytoma also described a decrease in PV immunoreactivity and numbers of AAC boutons in the peri-tumoral tissue (Marco et al., 1997), in line with other findings. Altogether, these studies reported a decrease in PV+ AACs' cartridges and an abnormal morphology of the remaining AACs. However, these results were only qualitative with no quantification of this apparent loss, which may also correspond to a decrease in PV expression. Despite the lack of quantification, these findings are consistent with previous experimental work showing degeneration of axonal boutons contacting the AIS at epileptic foci in the somatosensory cortex in a monkey model of focal epilepsy following intracerebral administration of alumina gel (Ribak, 1985).

#### Hippocampus

Studies on the human hippocampus of patients with EPI or animal models of temporal lobe EPI reported different alterations depending on the subregion studied and on the level of hippocampal sclerosis.

##### Cornu Ammonis

Wittner et al. (2005) found no change in synaptic coverage of pyramidal cells' AIS in human non-sclerotic Cornu Ammonis (CA) fields, in contrast to their somatic innervation. In sclerotic CA, these authors observed a decreased synaptic coverage of degenerating AISs, and a lack of AAC terminal staining for PV, suggesting a degeneration of AACs' cartridges. In the healthy (del Rio and DeFelipe, 1997) and mildly sclerotic CA1 region (Wittner et al., 2002), the AIS of pyramidal cells are innervated by CB-labeled boutons, which disappear in strongly sclerotic tissue, where pyramidal cells cannot be seen (Wittner et al., 2002). Similarly, in sclerotic hippocampal samples, a global loss of PV+ terminals has been described, with some PV+ cartridges remaining, of hypertrophic appearance (Arellano et al., 2004). These results collectively point toward a deficit in AAC innervation of pyramidal cells paralleling principal neuron degeneration in hippocampal sclerosis.

In the non-sclerotic Cornu Ammonis (CA), a study in rats reported that PV+ AAC boutons contacting pyramidal cells' AIS were intact and therefore resisted to kainic acid-induced seizures, in contrast to alterations in axo-somatic basket profiles (Best et al., 1994). On the contrary, in a rat model of chronic epilepsy through injection of pilocarpine, a decrease in the synaptic coverage of AIS has been observed (Dinocourt et al., 2003).

Finally, in a single physiological study using a model of interictal epileptiform discharge in CA3 slices of mice, AACs undergo increase in their firing with epileptiform discharges, suggesting an active role of AACs in generating epileptiform activity (Karlócai et al., 2014).

##### Dentate gyrus

In the sclerotic dentate gyrus (DG) of patients with temporal lobe EPI, the morphological “complexity” of PV+ and CB+ AACs' cartridges has been found to be increased, with more boutons and axonal rows per cartridge (Arellano et al., 2004). This increased complexity of AACs' cartridges could explain the findings of increased synaptic coverage of granule cells' AIS in epileptic patients (Wittner et al., 2001). Interestingly, the increase in AACs' cartridge complexity appeared to parallel the severity of sclerosis in the human DG (Wittner et al., 2001). The most recent work on human brain tissue found that the density of AACs' PV+ boutons is preserved in epileptic DG without sclerosis, and decreased in sclerosis (Alhourani et al., 2020). However, the density of VGAT+/GAD67+ boutons (i.e., putative axo-axonic terminals) was similar to controls in both cases (Alhourani et al., 2020), suggesting that AACs' terminals might be preserved, and PV expression decreased. In addition, GAD67 and VGAT expression levels were increased in AACs' terminals in sclerotic tissue, suggestive of a compensatory mechanism. Finally, axo-axonic boutons were found intact in DG in EPI modeled with either perforant-path electric stimulation or kainic acid administration to induce seizures in rats (Sloviter et al., 2003).

Overall, these findings indicate that the DG and the CA region follow opposite directions in hippocampal sclerosis, with reduced AIS synaptic coverage in CA, and preserved innervation by more complex cartridges of surviving DG granule cells. On the other hand, AACs appear to be preserved in the non-sclerotic hippocampus. A general finding in human studies is the reduced level of PV expression in histologically abnormal tissue.

### Schizophrenia

Thirteen papers were included (Figure 2), which reported molecular and functional alterations in AACs associated with SCZ (Table 2). Eight of them were post-mortem brain tissue analysis using immuno-histochemistry. The remaining five used animal models of SCZ.

**Table 2:** Summary of studies on axo-axonic cells in schizophrenia.

**References**	**Model(s)**	**Population characteristics (ratio M:F; age)**	**Area(s) studied**	**Methods**	**Variable(s) studied**	**Main findings**	**Comments**
Woo et al. (1998)	post-mortem human brains from patients with SCZ	10:5; 53.6 ± 13.0 (SD; SCZ), 10:5; 53.7 ± 12.4 (psychiatric controls); 10:5; 53.9 ± 13.8 (non-psychiatric controls)	BA9 and BA46, all layers	GAT-1 and calretinin IHC, light microscopy	Density of GAT-1+ cartridges	The density of GAT-1+ cartridges is decreased by 40% in SCZ vs. both control conditions. No variation in the mean density of GAT-1+ boutons nor in calretinin-IR boutons in layers 2-3a of BA9.	No variation in mean cartridge length, nor in PFC volume. Subjects treated with antipsychotics without SCZ had higher cartridge densities than in subjects with SCZ
Pierri et al. (1999)	Post-mortem human brains from patients with SCZ	20:10; 51.6 ± 13.0 (SCZ), 20:10; 51.6 ± 13.3 (psychiatric controls); 20:10; 52.1 ± 13.8 (non-psychiatric controls)	BA46, layers 2–3a, 3b−4, and 6	GAT-1 IHC, light microscopy	Density of GAT-1+ cartridges	The density of GAT-1+ cartridges is decreased in SCZ: - layers 2–3a 27% (vs. both controls) - layer 3b−4: 31.5 and 19.4% (vs healthy and psychiatric controls, respectively) - layer 6 (not statistically significant)	No variation in mean cartridge length, nor in PFC volume/cortical thickness. Higher cartridge density in patients treated with antipsychotic medication and increased cartridge density following haloperidol treatment in macaque.
Kalus et al. (1999)	Post-mortem human brains from patients with SCZ	3:2; 60.2 ± 20.6 (SCZ) 3:2; 66.0 ± 23.6 (non-psychiatric controls)	BA24c, layers 5 and 6	PV IHC, light microscopy	Density of PV+ cartridges	The density of PV+ cartridges is *increased* in SCZ by 59% in layers 5 and 6 of the ACC.	Small cohort. Only study focused on the deep layers (layer 5 in particular) and on the ACC
Volk et al. (2002)	Post-mortem human brains from patients with SCZ	9:5; 53 ± 8 (SCZ), 9:5; 53 ± 8 (MDD controls); 9:5; 52 ± 9 (non-psychiatric controls)	BA46, layers 2–3a	GABA-A Rα2 IHC, light microscopy	Density of α2-labeled AIS	The mean number of α2-labeled AIS is increased by 113% in SCZ. The densities of GAT-1+ cartridges and of α2-labeled AIS are inversely correlated.	Same cohort and techniques as Pierri et al. (1999). No variation in mean AIS length, nor in PFC volume. No effect of medication in the SCZ group.
Konopaske et al. (2006)	Post-mortem human brains from patients with SCZ	6:8; 48.3 ± 8.8 (SCZ), 6:8; 48.1 ± 7.1 (non-psychiatric controls)	BA42 (associative auditory cortex), layers 2–3a and 6	GAT-1 IHC, light microscopy	Density of GAT-1+ cartridges	No statistically significant (9.8%) decrease in the density of GAT-1+ cartridges in the auditory cortex.	Subjects drawn from the cohort and techniques of Pierri et al. (1999). No verification of variation in cartridge length or cortical volume
Cruz et al. (2009)	Post-mortem human brains from patients with SCZ	9:5, 52.6 ± 8.5 (SCZ); 9:5, 52.4 ± 8.6; (control with no mental disorder) 9:5, 53.0 ± 7.6 (MDD)	BA46, layers 2-3a and 6	Ankyrin G and βIV spectrin IHC	Density of ankyrin-G+ AIS and βIV-spectrin+ AIS in SCZ	decreased density of ankyrin-G+ AIS by 19 and 15% in superficial in cortical layers 2/3a in SCZ compared to control and MDD groups, respectively, but not in the deep (L6) cortical layer. No differences for βIV spectrin.	Same cohort and techniques as Pierri et al. (1999) and Volk et al. (2002). No variation in mean AIS length, nor in cortical volume.
Rocco et al. (2016)	Post-mortem human brains from patients with SCZ	14:6; 45 ± 7 (SCZ), 14:6; 46 ± 9 (non-psychiatric controls)	BA9, layers 2–6	VGAT and GAD67 IHC, light microscopy	VGAT and GAD67 protein levels in interneurons boutons	VGAT and GAD67 protein levels in VGAT+ AAC boutons do not differ between SCZ and controls, in contrast to other interneurons.	The total number of VGAT+ cartridges or AAC boutons in each group is not reported (but see Rocco et al., 2017). No statistically significant effect of antipsychotic medication on the global result.
Rocco et al. (2017)	Post-mortem human brains from patients with SCZ	14:6; 45 ± 7 (SCZ), 14:6; 46 ± 9 (non-psychiatric controls)	BA9, layers 2–6	CB, GAD67 and VGAT IHC, light microscopy	Density of VGAT+ and VGAT+/CB+ cartridges	Higher density of VGAT+ cartridges (+21%) in layer 2-superficial layer 3 of SCZ vs. control PFC. Accounted for by a 2.7 increase in CB+/VGAT+ cartridge density in layer 2. No difference in deeper layers in VGAT+/CB+, VGAT+/CB- cartridge density, or VGAT levels in AAC terminals	Same cohort and techniques as in Rocco et al. (2016). No statistically significant effect of antipsychotic medication on the global result. No measurement of GAT1 as a replication of previous results. No assessment of variations in cartridge length or cortical volume.
Morrow et al. (2007)	PCP-treated monkey	4:0; adults (PCP-treated) 4:0; adults (controls).	Walker's area 46 (homologous to BA46), all layers	PV IHC, light microscopy	Number of PV+ cartridges across the cortex	Decreased number of PV+ axo-axonic structures by about 40% in layers 2/3 of PCP-treated monkeys. No change in spatial distribution across layers. No change in numbers or distributions of PV+ and CR+ cells.	A decrease in PV expression cannot be excluded. The exact age of monkeys is unknown.
Bloomfield et al. (2008)	Isolation-reared rats	IHC: 4:0M; 20-week (socially reared); 3:0; 20-week (isolation reared) PPI testing only: males (*n* unknown), P84 for both	mPFC (anterior cingulate, prelimbic and infralimbic)	GAT-1 IHC, PPI testing	Total number of GAT-1+ cartridges and PPI	Reduced number of GAT-1+ cartridges by 36% in the ventral prelimbic cortex of isolation reared rats. No difference in other mPFC divisions (anterior cingulate, dorsal prelimbic, infralimbic).	A decrease in GAT-1 expression cannot be excluded. No measure of cortical volume, no verification of cartridge length. No direct link established between behavioral and morphological findings
Del Pino et al. (2013)	Mutant mouse model (conditional ErbB4 mutants = Lhx6-Cre; Erbb4 F/F mice)	IHC and biochemistry: P30 mice. For *in vitro* electrophysiology: P20-22 mice. For *in vivo* electrophysiology on anesthetized mice: P2-3-month male mice (number not specified).	Hippocampus and PFC	vGluT1, GABA-A Rα2, ankyrin-G IHC. *in vitro* and *in vivo* electrophysiological recordings + behavioral assays (activity cages, elevated-plus maze,	Impact of a conditional ErbB4 KO in PV interneurons on AAC's morphology and function, and on behavior	The soma and dendrites of neocortical AACs lacking Erbb4 receive significantly less VGlut1+ terminals than control cells. The density of boutons in AACs cartridges and of GABAAa2 clusters at AIS is decreased. Mutant mice showed synaptic defects in particular increased	Extensive study which analyzed from the molecular to behavioral impact of the mutation. The manipulation of ErbB4 is not specific for AAC, also affecting basket cells. Missing details regarding animals gender and age.
				Y maze, marble burying, three-chambered social test, nesting, PPI)		excitability (PV+ fast-spiking neurons and pyramidal cells), abnormal synchrony, increased oscillatory activity- increased locomotor activity, abnormal emotional responses, impaired social behavior and impaired cognitive function.	
Yang et al. (2019)	Mutant mouse models (cKO mice = Nkx2.1CreER;erbb4^−/−^ and ErbB4 knockdown mice = Nkx2.1CreER;LSL-Flpo with Flp-dependent shRNA targeting ErbB4 in mPFC	*In vitro* electrophysiology: P21-30 or 2 weeks following AAV injections (made at P45-50). 2–3-month-old males for behavior testing. P30 for western blot.	mPFC, layers 2/3	PV, ErbB4, vGluT1 and ankyrin G IHC + *in vitro* electrophysiology + behavioral assays (open field, elevated-plus maze, PPI test, automated radial arm maze, three-chamber social interaction test)	Impact of ErbB4 KO in mPFC AACs on their morphology and functions	Immunohistochemistry: cKO : decrease in the density of AACs' boutons on AISs, decreased density of vGluT1+ boutons on AAC somata; knock-down: no data. Electrophysiology: cKO: decreased frequency and amplitude of mEPSCs in AACs, decreased amplitude of mIPSCs in pyramidal neurons; knock-down: decreased frequency and amplitude of mEPSCs in AACs, decreased frequency of mIPSCs in pyramidal neurons. Behavior: cKO: impaired PPI, hyperactivity, working memory deficits normalized by an agonist of GABA_A_-Rα2 receptors agonist; knock-down: impaired working memory, impaired social novelty recognition (no effect on PPI/locomotion).	The gender or the number of mice for each experiment is unknown.
Fujikawa et al. (2021)	Intraperitoneal Ketamine injections (daily for 7 days) in wild type (C57BL/6J) mice; control= saline	Not reported	Dorsal hippocampus	SATB1, PV, NPY, SST, (HNK-1) glycan (Cat-315 antibody) IHC	Density of PV+ cells and cell types relative to Cat-315 expression	Decreased density of Cat-315- AACs and no change in Cat-315+ AACs in the ketamine group. Decreased density of Cat-315+/PV+ neurons in stratum oriens and stratum pyramidale; no change in Cat-315-/PV+ neuron density; increased Cat-315+/PV- density in the ketamine group (primary result).	Age of the mice unknown Many cell types affected by ketamine in various ways No causal links established in ketamine/Cat-135/interneuron type interactions.

AAC, axo-axonic cell; AAV, adeno-associated virus; ACC, anterior cingulate cortex; AIS, axon initial segment; BA, Brodmann area; cKO, conditional knockout; eGFP, enhanced green fluorescent protein; Errb4, Erb-B2 receptor tyrosine kinase 4; GABAR, gamma-aminobutyric acid receptor; GAT-1, GABA transporter 1; HNK-1, human natural killer-1 (glycan); H-S, hippocampo-septal projection interneurons; IHC, immunohistochemistry (also used here for immunofluorescence for simplicity); MDD, major depressive disorder; mPFC, medial prefrontal cortex; NPY, neuropeptide Y; O-LM, oriens-lacunosum moleculare (interneuron); PCP, phencyclidine; PPI, pre-pulse inhibition; PV, parvalbumin; Px, postnatal age, in days; SATB1, special AT-rich sequence-binding protein-1; SCZ, schizophrenia; SD, standard deviation; SOM/SST, somatostatin; VGLUT1, vesicular glutamate transporter 1.

#### Post-mortem studies

Except for one study (Konopaske et al., 2006), all post-mortem studies were performed on the prefrontal cortex (PFC), which activity is altered in SCZ (Minzenberg et al., 2009). All reported pre- and/or post-synaptic alterations of AACs to pyramidal cell connections, albeit with conflicting conclusions.

##### Pre-synaptic alterations

The number of axo-axonic boutons and cartridges has been reported to be altered to various degrees in SCZ. The first article of this kind reported a 40% decrease in the density of GAT-1+ AACs' cartridges in SCZ compared to controls (Woo et al., 1998). This decrease later appeared to concern mainly layers 2–3 of the PFC in patients with SCZ, compared to controls (Pierri et al., 1999). It was then interpreted as reflecting either a decrease in cartridge density or reduced levels of GAT-1. Interestingly, this alteration displayed some degree of specificity for the PFC, since using the same approach, no significant decrease in GAT-1+ AACs' cartridges was found in the auditory cortex (Konopaske et al., 2006), and an increased density of PV+ AACs' cartridges was reported in layer 5/6 of the anterior cingulate cortex of patients with SCZ (Kalus et al., 1999). In contrast to bouton density, the expression levels of presynaptic proteins including GAD67 or VGAT were found to be preserved in SCZ subjects (Rocco et al., 2016).

In fact, the group that reported reduced PFC axo-axonic innervation in SCZ (Woo et al., 1998; Pierri et al., 1999) recently found that the number of axo-axonic boutons was unaltered in SCZ, using VGAT (instead of GAT-1) as a presynaptic marker (Rocco et al., 2017). Moreover, the density of AACs' CB+ cartridges is increased in layer 2 of PFC (Rocco et al., 2017), with no change in CB-negative cartridges density or VGAT expression levels in SCZ. Therefore, the suspected defect in AACs' cartridges (Woo et al., 1998; Pierri et al., 1999) likely reflected a decreased expression of GAT-1. Importantly, these findings did not appear to be a consequence of gender status, nicotine use, benzodiazepines and/or sodium valproate, antidepressants, or antipsychotics when accounted for in statistical analyses (Rocco et al., 2016, 2017).

##### Post-synaptic alterations

The density of AISs expressing the GABA_A_ receptor α2 subunit (GABA_A_-Rα2) is increased in PFC from patients with SCZ, and negatively correlated with the numbers of GAT-1+ AACs' cartridges (Volk et al., 2002). This result was interpreted as a compensatory mechanism, with increased GABA_A_-R α2 expression balancing a reduced number of GABAergic terminals- and in turn a likely reduced release of GABA. However, as Lewis and collaborators later showed, the number of GABAergic terminals was preserved (Rocco et al., 2017). Finally, a decrease in the density of Ankyrin-G+ AISs in the absence of β-spectrin+ (another AIS scaffold) has been reported in the PFC of patients with SCZ (Cruz et al., 2009). This result suggests a decreased expression of Ankyrin-G at AISs in SCZ, and potential defects in function since Ankyrin-G is a key scaffold protein for axo-axonic synapses at the AIS (Tai et al., 2019). Overall, evidence is limited regarding the involvement of AACs' postsynaptic changes in SCZ.

#### Animal models of schizophrenia

Studies on animal models of SCZ have provided evidence for structural alterations in AACs and for links between structural findings and functional or behavioral deficits.

Regarding structural studies, a monkey model of SCZ generated with repeated treatments with phencyclidine displayed a 40% decrease in the number of prefrontal PV+ axo-axonic structures (Morrow et al., 2007). In the isolation-reared rat model of SCZ, no global differences in the total number of GAT-1+ cartridges were found in the rat PFC, but a local decrease by 36% was identified in the ventral prelimbic region relative to control (Bloomfield et al., 2008). Lastly, in a ketamine model of SCZ in mice, interneurons were studied with regards to the presence of perineuronal nets detected with the marker Cat-315; (Fujikawa et al., 2021). The number of Cat-315–/PV+ AACs (identified with PV expression in the absence of neuropeptide Y or SATB1 expression) were fewer in ketamine-treated mice compared to controls, while Cat-315+/PV+ AACs were not affected (Fujikawa et al., 2021).

More direct evidence of AAC implication in SCZ has been provided by the conditional KO of *ERBB4* (a SCZ risk-gene) in medial ganglionic eminence-derived interneurons (comprising PV+ cells) in mice (Del Pino et al., 2013). Among synaptic defects, Del Pino et al. found a decreased density of PV+ axo-axonic boutons and a decreased density of GABA_A_-R α2 clusters at the AIS contrary to human post-mortem results of Volk et al. (2002). AACs also received fewer excitatory synapses, as measured anatomically and physiologically with reduced miniature excitatory postsynaptic currents frequencies *in vitro*. These defects were accompanied by global increase in excitability, both of PV+ interneurons and of pyramidal cells *in vitro*. *In vivo*, this resulted in more frequent population spike occurrence in the DG, increased hippocampal oscillation power and decreased synchrony between brain regions (Del Pino et al., 2013). The latter oscillatory pattern is reminiscent of abnormalities found in SCZ (Hunt et al., 2017). Furthermore, mutant mice showed behavioral impairments consisting of increased locomotor activity, reduced anxiety, impaired working memory and reduced social interactions (Del Pino et al., 2013). However, the behavioral analysis lacked specificity for AACs. With the implementation of a novel mouse line to target selectively AACs by temporal tagging of Nkx2.1-expressing progenitors (Nkx2.1CreER; He et al., 2016), these results were confirmed by performing KO of *ERBB4* selectively in AACs (Yang et al., 2019). The density of AACs was preserved, while the density of their synaptic boutons contacting the AIS was decreased. AACs also received less glutamatergic innervation (Yang et al., 2019), in agreement with previous findings on MGE-derived interneurons (Del Pino et al., 2013). Consistently, the frequency of miniature inhibitory postsynaptic currents was reduced in pyramidal neurons. Behaviorally, mice displayed increased locomotor activity, reduced paired-pulse inhibition, impaired working memory and altered social behavior (Yang et al., 2019), in line with the previous study (Del Pino et al., 2013). Behavioral deficits were rescued by systemic or intra-PFC infusion of the partial GABA-A Rα2 agonist L-838417 (Yang et al., 2019). These results were recapitulated in the Nkx2.1CreER conditional knock-down of *ERBB4* in AACs restricted to the PFC, suggesting the results were not the consequence of developmental compensatory mechanisms, and that prefrontal AACs might be implicated in schizophrenia (Yang et al., 2019).

Overall, these results are consistent in showing that pyramidal neurons innervation by AACs is reduced in animal models of SCZ, and that such alterations might directly underlie pathological phenotypes via reduced functional inhibition at the AIS and perturbed brain rhythms.

### Autism spectrum disorders

Three articles were found which reported on AACs in ASD (Figure 3), studying post-mortem tissue from PFC (Table 3).

**Table 3:** Summary of studies on axo-axonic cells in autism spectrum disorders.

**References**	**Model**	**Population characteristics (ratio M:F; age range)**	**Area(s) studied**	**Methods**	**Variable(s) studied**	**Main findings**	**Comments**
Ariza et al. (2018)	Post-mortem human brain tissue from ASD patients	10:0; 7–48 (ASD; 9:1; 6–56 (control)	BA9, BA46, BA47	PV and VVA IHC	Percentage of AACs (defined as PV+/VVA–) among the total number of cells	Decreased percentage of PV+ AACs in ASD, by 65.1% in BA46, by 39.1% in BA47 and by 40% in BA9	Except for one control case, subjects were all males. Only PV+ AACs were studied. Data are expressed in percentages to avoid the introduction of error due to differential tissue shrinkage, but no information about the total number of neurons is provided.
Hong et al. (2020)	Post-mortem human brain tissue from ASD patients	19:1; 6–56 (ASD); 19:1; 7–56 (control)	BA9, BA46, BA47 (layers 3 and 5)	GABA-A Rα2 and neurofilament IHC	Percent area of AIS occupied by GABA-A Rα2 in ASD brain tissue	The percent area of GABA-A Rα2 staining in pyramidal cells AIS is decreased in layer 3 by 61% in BA9 and by 54% in BA47 in ASD (no difference in layer 5). No difference in BA46.	Manual delineation of AIS, but blinding of phenotype.
Amina et al. (2021)	Post-mortem human brain tissue from ASD patients	11:0; 7–23 (ASD); 7:4; 7–24 (control)	BA9, BA46, BA47	GAT-1 IHC	Number of GAT-1+ cartridges per defined area (3 mm band from pia to white matter)	Decrease in the total number of GAT1+ cartridges in ASD	The definition of areas analyzed is not normalized by area: potential confounding factor if surface was decreased in ASD samples.

AAC, axo-axonic cell; ASD, autism spectrum disorder; BA, Brodmann area; GABA-A R, type A GABA receptor; GAT-1, GABA transporter 1; PV, parvalbumin; VVA, Vicia villosa agglutinin.

To distinguish between PV+ AACs and PV+ basket cells, Ariza and colleagues used double immunohistochemical labeling of PV and *Vicia Villosa* lectin and the PV+/VVA– phenotype as a proxy for AACs (Ariza et al., 2018). They found a 40–65% decrease in the absolute number of PV+ AACs while the number of PV+ basket cells was not significantly reduced (Ariza et al., 2018). The absolute number of GAT-1+ cartridges was found to be decreased by 40–60% across prefrontal areas by the same group (Amina et al., 2021). This result may support the hypothesis that prefrontal AACs' density is reduced in ASD as opposed to PV or GAT-1 immunoreactivity.

Post-synaptic alterations were also reported for AACs, with a reduction in the AIS surface covered by GAB_A_A-Rα2 subunits in upper cortical layers of samples from patients with ASD (Hong et al., 2020). This result suggests that presynaptic loss of AACs is not compensated for by an increase in postsynaptic expression of GABA_A_-Rs in ASD.

## Discussion

**Figure 4:**
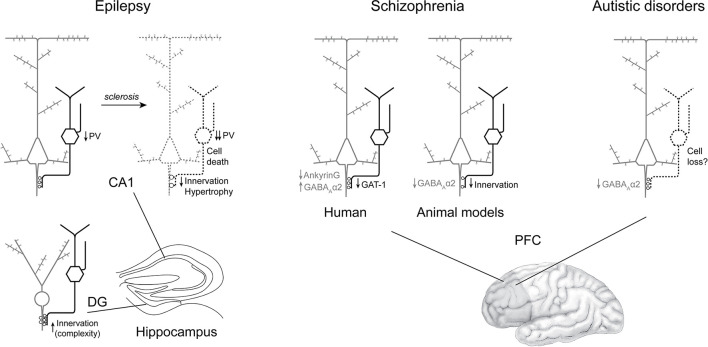
Summary of axo-axonic cells alterations consistently found in epilepsy, schizophrenia and autism spectrum disorder. In epilepsy **(left)** without sclerosis, AACs are spared in both CA1 (with reduced PV expression) and dentate gyrus (with findings of increased axo-axonic cartridge complexity). In the sclerotic CA1, PV expression, axo-axonic cell density and axo-axonic innervation appear altered, paralleling pyramidal cell loss. In brain samples from patients with schizophrenia **(middle)**, the density of AACs seems preserved, while in their synaptic boutons, GAT-1 is downregulated. At the postsynaptic level, there are more GABA_A_-Rα2 clusters. In contrast, in animal models, axo-axonic innervation is reduced along with GABA_A_-Rα2 cluster density. In ASD **(right)**, preliminary evidence point toward a reduced density of GABA_A_-Rα2 clusters, and a possible AAC loss.

The aim of this work was to provide a systematic review of AACs' alterations in neuropsychiatric disorders. Namely, articles on AACs in EPI, SCZ and ASD were studied. Review of the 32 articles relevant to this topic point to alterations in AAC to AIS synapses in brain disorders (summarized in Figure 4). Previous articles have reviewed AACs' function, and have debated their implication in disease, including recent publications (Wang et al., 2016; Gallo et al., 2020; Juarez and Martinez Cerdeno, 2022; Jung et al., 2022). However, to the best of our knowledge, none of the previous reviews has followed a systematic procedure, precluding a thorough analysis of AAC dysfunction in disease. Our review article thus provides a comprehensive summary of AACs in three main neuropsychiatric disorders, in a systematic format, for the first time. Below, we briefly summarize and articulate the main findings, before discussing their limitations and suggesting some perspectives.

### Main findings

Overall the systematic review of AACs' implication in EPI, SCZ, and ASD revealed some consistency, but also many contradictory findings, especially in human tissue.

In EPI, there appears to be region-specific alterations. In hippocampus, defects vary depending on the region, and with the amount of sclerosis severity. A consistent finding is reported in the sclerotic CA, with PV+ and CB+ AACs' cartridges displaying decreased density and PV expression levels, while no clear phenotype emerged from studies in non-sclerotic tissue. On the contrary, in the epileptic DG, AACs seem to be preserved, and display increased arborization complexity paralleling sclerosis. In contrast, an earlier study reported a qualitative decrease of GAT-1 immunoreactivity in the DG, including at the border between the granule cell layer and the subgranular hilus, where AISs are located (Sayin et al., 2003). Surgical samples have been reported to be heterogeneous within and across subjects of individual studies (e.g., Arellano et al., 2004), reflecting biological variability, rather than differences in techniques applied for fixation or tissue processing. Thus, it is likely that the small sample sizes and heterogeneity in the subjects analyzed may partly account for the general lack of consistency in the studies reviewed here. The loss of AACs in the sclerotic tissue might contribute to pathological activities by loss of inhibition, or by GABAergic excitation in this condition (e.g., Hristova et al., 2021, but see Krook-Magnuson et al., 2013), but could also merely reflect a global cell loss, proportional to pyramidal cell death. Intriguingly, alterations in AACs did not mirror epileptiform activities, when the latter were recorded in patients before surgical removal of the samples subsequently analyzed (DeFelipe et al., 1993; Marco et al., 1997). This suggests that AACs might not play an active role in epileptogenesis.

In SCZ, initial studies pointed to the loss of AACs' cartridges in human tissue. However, complementary analyses revealed that AACs' terminals were preserved, indicating that initial findings reflected the inability to detect molecular markers in axo-axonic boutons because of low expression levels (Rocco et al., 2017). In contrast, the density of CB-expressing cartridges may be increased (Rocco et al., 2017) in superficial layers of the PFC in SCZ, the meaning of which is uncertain. Expression of postsynaptic GABA_A_-Rα2, on the other hand, may be increased, the causes and consequences of which also remain to be established. Paradoxically, in animal models, consistent reductions of AIS innervation by AACs were found, along with decreased GABA_A_-Rα2 expression postsynaptically and decreased glutamatergic innervation presynaptically (Del Pino et al., 2013; Wang et al., 2016). Notably, one of the hypothesis on the onset of schizophrenia is a decrease of NMDA transmission, which implicate PV+ interneurons (e.g., Gawande et al., 2023), and perhaps AACs. In the animal model of *ERBB4* KO in AACs, cognitive alterations were improved by a GABA_A_-Rα2 agonist (Yang et al., 2019). Interestingly, in an open label trial, the GABA_A_-Rα2-3 selective agonist MK-0777 improved cognitive function in patients with SCZ (Lewis et al., 2008). However, a subsequent randomized controlled trial failed to demonstrate any benefit (Buchanan et al., 2011). There are many potential explanations to these discrepancies between human and animal studies. For instance, the animal models employed by these authors relied on monogenetic alterations that may not reflect the full and complex spectrum of polygenic alterations found in schizophrenia (Trubetskoy et al., 2022). To date, it remains unclear how AACs are altered in SCZ, and what could be the direct contribution of these alterations to clinical symptoms.

Research on AACs' implication in ASD is still recent, and only three articles were found in the inclusion period, reporting a reduced number of AAC somata and cartridges (Ariza et al., 2018; Amina et al., 2021) and a decrease in postsynaptic GABA_A_-Rα2 at the AIS in the PFC of subjects with ASD (Hong et al., 2020). The indirect detection of AACs and cartridges using molecular markers as proxies calls for caution, as exemplified by the case of SCZ (and see below). The decreased density of GABA_A_-Rα2 at the AIS (Hong et al., 2020) is also in contrast with findings in SCZ (Volk et al., 2002), with unknown functional consequences. Despite the paucity of the literature, these initial results suggest a potential implication of the axo-axonic synapse in ASD pathophysiology.

It is intriguing that EPI, SCZ and ASD implicate overlapping brain regions, which contain relatively high densities of AACs (Inda et al., 2009). Those disorders are linked to neurodevelopmental abnormalities, while AACs have a peculiar, late developmental pattern compared with other interneurons (Taniguchi et al., 2013). Moreover, AACs undergo structural and functional maturation until young adulthood (Rinetti-Vargas et al., 2017; Steinecke et al., 2017), a period at risk of SCZ or EPI occurrence. Comorbid EPI and psychotic disorders or ASD, are highly prevalent in clinical populations (Clancy et al., 2014; Tye et al., 2018). As a result, one may speculate that common pathophysiological principles may be at stake and implicate AACs. To test this hypothesis, it would be interesting to investigate alterations of AACs in brain samples from subjects with comorbid disorders. Moreover, animal studies suggest that AACs might be implicated in other neurodevelopmental disorders. Prenatal exposure to cocaine results in decreased density of AAC cartridges in the PFC (Morrow et al., 2003). In a mouse model of Down syndrome, AACs displayed aberrant innervation of AIS with increased number of cartridges, cartridge length, bouton size and amount of GABAergic inhibition in electrophysiological recording (Liu et al., 2023). Human counterparts of these findings are currently lacking. In summary, AACs may bear common and direct pathogenic factors across EPI, SCZ and ASD, but the focus of the review on this topic may also artificially inflate this hypothesis.

### Limitations and perspectives

The first main limitation is the result of indirect strategies to study AACs in human brain samples. Most of the histological methods applied to human tissue have so far relied on molecular markers, be it to infer the density of AACs to pyramidal cells synapses, or to evaluate the density of AACs. Clear limitations of this strategy are highlighted in the following examples. First, in SCZ, the reduction of GAT-1+ cartridges later appeared to reflect a reduction in protein levels rather than the loss of AACs (Rocco et al., 2017). Second, PV+ cartridges have been widely used as a proxy for axo-axonic synapses in EPI studies. However, the same studies consistently reported a global reduction of PV immunoreactivity in epileptic tissue (Wittner et al., 2001; Arellano et al., 2004), which might have contributed to the observation of reduced AAC cartridges density. Moreover, a significant number of AACs may be devoid of PV in the healthy brain (Taniguchi et al., 2013; Tasic et al., 2018). The case of increased CB+ axo-axonic cartridges density in SCZ (Rocco et al., 2017) or in EPI (Arellano et al., 2004) is intriguing in this context. Third, in an ASD study, the absence of perineuronal nets around PV+ cells was used to identify AACs (Ariza et al., 2018), while perineuronal nets have been extensively found around hippocampal AACs (Fujikawa et al., 2021). Alterations in molecular content of AACs may indeed be the consequence of compensatory or even confounding factors, including pharmacological treatments. In addition, most Human studies were published by one or two research groups in each disorder, calling for replication studies.

The second main limitation in knowledge is the lack of experimental evidence to causally link structure and function. Indeed, most of the articles reviewed here were reports of histopathological studies in Human, which are, by essence, correlative and cannot provide mechanisms. Animal models are instrumental to understand pathogenesis and suggest causal links between cellular alterations and symptoms. However, only very few studies were found on this topic, and data is still lacking at all levels: cells (synaptic transmission), network (assemblies, oscillations), and behavior. We have highlighted three important functional studies (Konopaske et al., 2006; Del Pino et al., 2013; Yang et al., 2019), also showing discrepancies in histological findings with human data. Animal models will thus need to be cross validated with Human data in order to improve translation. One interesting pathway is the reduced density of GABA_A_-Rα2 at the AIS, as suggested by animal studies in SCZ (Del Pino et al., 2013, but see Volk et al., 2002) and clinical samples in ASD (Hong et al., 2020). In addition, reduced levels of GABA_A_-Rα2 at the AIS (possibly from AACs) induce greater seizure susceptibility in mice (Hines et al., 2018). GABA_A_-Rs containing the α2 subunit show fast activation and slow deactivation kinetics (Lavoie et al., 1997). Therefore, the loss of this postsynaptic partner of AACs might induce a strong hyperexcitability in principal neurons. Until recently, AACs studies have been limited by the lack of specific genetic markers. Using single-cell RNA sequencing, such markers have been identified, including PTHLH, Uncb5 and Vipr2 (Paul et al., 2017; Tasic et al., 2018). As a result of these discoveries, genetic targeting of cortical AACs is now accessible in animal models using Nkx2.1CreER (He et al., 2016) or Vipr2-Cre mouse lines (Tasic et al., 2018) for the neocortex, or using Unc5b-2A-CreER mice for the hippocampus (Dudok et al., 2021). It is beyond the scope of this work to extensively review this, but recent work have started to unravel AACs functions in physiological conditions using these transgenic animals (e.g., Lu et al., 2017; Dudok et al., 2021; Schneider-Mizell et al., 2021; Jung et al., 2023; Seignette et al., 2023). These showed that AACs only weakly inhibited principal neurons' firing, suggesting that alterations of AACs in disease might not result in gross excitation/inhibition imbalance. In fact, AACs seem to ensure excitation homeostasis in cortical networks (Pan-Vazquez et al., 2020), a loss of which may have dramatic consequences on cortical function (e.g., overexcitation, increased local synchrony and reduced interregional coordination).

Direct examination of AACs is warranted, and may rely on complementary approaches. Detailed reconstructions of AACs at the cellular and ultrastructural levels following Golgi staining or semi-automated electron microcopy (e.g., Schneider-Mizell et al., 2021) would provide invaluable information. The study by Wittner et al. (2005) is noteworthy as being the only one in this review that systematically examined AIS innervation at the electron microscopic level regardless of PV content. Recently identified molecular markers of AACs will also prove useful in identifying AACs in human tissue (e.g., Alhourani et al., 2020). Moreover, targeting AACs with novel transgenic mouse lines represent an opportunity to directly evaluate whether a genetic mutation implicates AACs in generating a pathological phenotype (as in Yang et al., 2019). Targeting AACs for recording and opto- or chemo-genetic manipulations could also be implemented to study their implications in disease models constructed with non-genetic interventions (e.g., pharmacological, lesional). These experiments are expected to provide key evidence to causally link axo-axonic cells to brain disorders.

## Conclusion

Although observations made in brain tissue from human subjects and in animal models indicate that axo-axonic cells may be altered in epilepsy, schizophrenia, and autism spectrum disorders, the literature is limited and mainly inconsistent, precluding clear conclusions to be drawn.

By joining clinical research and refined preclinical models, we hope that the coming years will bring significant clarification and progress in the field of AACs in disease.

## Data availability statement

The original contributions presented in the study are included in the article/Supplementary material, further inquiries can be directed to the corresponding author.

## Author contributions

JV and AE: literature search and screening. JV, AE, and TB: article selection. JV, AE, CL, and TB: literature synthesis. JV, AE, CL, FL, BA, CH, YH, and TB: manuscript writing and revisions. TB: supervision of the project. All authors contributed to the article and approved the submitted version.
