# Supplementary material for: Axo-axonic cells in neuropsychiatric disorders: a systematic review
Source: Front Cell Neurosci. 2023 Jun 26;17:1212202. doi: 10.3389/fncel.2023.1212202 (PMC10330806; doi:10.3389/fncel.2023.1212202)
Supplement: Supplementary file 1 [file Table_1.docx]

| Study (author and year) | Model(s) | Population characteristics (ratio M:F; age range) | Area studied | Methods | Variable studied | Main findings | Reason of exclusion |
| --- | --- | --- | --- | --- | --- | --- | --- |
| Kisvárday et al., 1986 | Biopsy tissue from patient affected by temporal lobe epilepsy | 1 male, aged 15 years old | Temporal cortex, likely layer 3 | Golgi staining, light and electron microscopy | Characteristics of **one impregnated AAC** | 269 cartridges identified, containing 3-12 boutons. One cartridge was making type II synapses to the AIS of a Golgi-impregnated pyramidal cell. This same AIS alo received numerous other type II synapses. | No report of AAC **alteration** |
| Sayin et al., 2003 | Twice daily kindling stimulation  (5 days per week) to evoke afterdischarge in perforant path and subsequent seizures | Adult Sprague Dawley rats (all males, age: N/A) | Hippocampus (dentate gyrus) | PV and GAT-1 IHC. | PV and GAT-1 staining variation between kindled rats experiencing spontaneous seizures and age-matched controls | No significant alteration in PV staining between kindled rats experiencing spontaneous seizures and age-matched controls.  **Reduction in GAT-1 staining in hippocampus** of rats experiencing >90 evoked seizures, “**particularly prominent along the border of the granule**  **cell layer and subgranular region of the hilus, which is the site of axon initial segments and spike initiation**”. | The decrease in GAT-1 staining is qualitative and **no unequivocal identification of AACs or AIS** by molecular or structural marker |
| Fujiwara-Tsukamoto et al., 2004 | Tetanic stimulation in the stratum radiatum | Wistar rats (sex: N/A, 20-30 days old) | Hippocampus (CA1) | Whole-cell patch clamp paired recordings | Electrophysiological responses of interneurons and pyramidal cells during evoked seizure-like afterdischarge | **A putative AAC exhibited strong bursting responses during evoked seizure-like afterdischarge**, synchronous with oscillatory activity in the simultaneously recorded pyramidal cell. The first discharge in the putative AAC preceded the oscillatory activity in the pyramidal cell | **No robust identification of AACs** (“*putative* chandelier interneuron”) |
| Wang et al., 2014 | Optogenetic activation of VGAT-expressing interneurons with a laserspritzer slectively at the AIS of pyramidal cells and epileptiform after-discharge induction | VGAT-ChR2-YFP mice (sex: N/A, age: 6-8 weeks old) | Piriform cortex (layer 2 and 3) | Ankyrin G, Na_v_1.2 and GAT-1 IHC. Confocal microscopy. Brain slice electrophysiology (laserspritzer activation of axo-axonic synapses and IPSCs recording in pyramidal cells, gramicidin-based perforated patch recordings) | Spatial distribution of AAS and electrophysiological properties of AAS onto AIS. | Axo-axonic synapses (AAS) found on AIS where Na_v_1.2 cluster.  Laserspritzer activation of AAS induce robust IPSCs induction throughout the entire length of the AIS. **Laserspritzer activation of AAS has a strong inhibitory effect on spike induction at the AIS. Laserspritzer activation of AAS also suppresses epileptiform afterdischarge *in vitro***. | No report of AAC **alteration** in EPI. |

**Supplementary table: Summary of studies that were excluded after discussion among authors**

*List of abbreviations*

AAC: axo-axonic cell; AAS: axo-axonic synapse; AIS: axon initial segment; CA1: *Cornu ammonis* 1 (hippocampal region); EPI: epilepsy; GAT-1: GABA transporter type1; IHC: immunohistochemistry; IPSCs: inhibitory post-synaptic currents; Na_v_1.2: voltage-gated sodium channels 1.2; PV: parvalbumin; VGAT-ChR2-YFP mice: vesicular γ-aminobutyric acid transporter (VGAT)–channelrhodopsin 2 (H134R) (ChR2)-enhanced yellow fluorescence protein (YFP) mice.

**References**

Fujiwara-Tsukamoto, Y., Isomura, Y., Kaneda, K., & Takada, M. (2004). Synaptic interactions between pyramidal cells and interneurone subtypes during seizure-like activity in the rat hippocampus. The Journal of physiology, 557(Pt 3), 961–979

Kisvárday, Z. F., Adams, C. B., & Smith, A. D. (1986). Synaptic connections of axo-axonic (chandelier) cells in human epileptic temporal cortex. Neuroscience, 19(4), 1179–1186.

Sayin, U., Osting, S., Hagen, J., Rutecki, P., & Sutula, T. (2003). Spontaneous seizures and loss of axo-axonic and axo-somatic inhibition induced by repeated brief seizures in kindled rats. The Journal of neuroscience : the official journal of the Society for Neuroscience, 23(7), 2759–2768.

Wang, X., Hooks, B. M., & Sun, Q. Q. (2014). Thorough GABAergic innervation of the entire axon initial segment revealed by an optogenetic 'laserspritzer'. The Journal of physiology, 592(19), 4257–4276.
